# Stillbirths and newborn deaths in slum settlements in Mumbai, India: a prospective verbal autopsy study

**DOI:** 10.1186/1471-2393-12-39

**Published:** 2012-05-30

**Authors:** Ujwala Bapat, Glyn Alcock, Neena Shah More, Sushmita Das, Wasundhara Joshi, David Osrin

**Affiliations:** 1 Society for Nutrition, Education and Health Action (SNEHA), Urban Health Centre, Chota Sion Hospital, 60 Feet Road, Shahunagar, Dharavi, Mumbai, 400017, Maharashtra, India; 2 UCL Centre for International Health and Development, Institute of Child Health, 30 Guilford St, London, WC1N 1EH, UK

**Keywords:** Verbal autopsy, Perinatal mortality, Stillbirth, Newborn death, Urban health, Mumbai, India, Slum

## Abstract

**Background:**

Three million babies are stillborn each year and 3.6 million die in the first month of life. In India, early neonatal deaths make up four-fifths of neonatal deaths and infant mortality three-quarters of under-five mortality. Information is scarce on cause-specific perinatal and neonatal mortality in urban settings in low-income countries. We conducted verbal autopsies for stillbirths and neonatal deaths in Mumbai slum settlements. Our objectives were to classify deaths according to international cause-specific criteria and to identify major causes of delay in seeking and receiving health care for maternal and newborn health problems.

**Methods:**

Over two years, 2005–2007, births and newborn deaths in 48 slum areas were identified prospectively by local informants. Verbal autopsies were collected by trained field researchers, cause of death was classified by clinicians, and family narratives were analysed to investigate delays on the pathway to mortality.

**Results:**

Of 105 stillbirths, 65 were fresh (62%) and obstetric complications dominated the cause classification. Of 116 neonatal deaths, 87 were early and the major causes were intrapartum-related (28%), prematurity (23%), and severe infection (22%). Bereavement was associated with socioeconomic quintile, previous stillbirth, and number of antenatal care visits. We identified 201 individual delays in 121/187 birth narratives (65%). Overall, delays in receiving care after arrival at a health facility dominated and were mostly the result of referral from one institution to another. Most delays in seeking care were attributed to a failure to recognise symptoms of complications or their severity.

**Conclusions:**

In Mumbai’s slum settlements, early neonatal deaths made up 75% of neonatal deaths and intrapartum-related complications were the greatest cause of mortality. Delays were identified in two-thirds of narratives, were predominantly related to the provision of care, and were often attributable to referrals between health providers. There is a need for clear protocols for care and transfer at each level of the health system, and an emphasis on rapid identification of problems and communication between health facilities.

**Trial registration:**

ISRCTN96256793

## Background

Three million babies are stillborn each year and 3.6 million die in the first month of life [1,2]. Stillbirth describes the death of an infant before complete delivery, at a gestation beyond 22 weeks (according to ICD-10), or 28 weeks (previous classifications, and common usage in low-income settings) [3]. Neonatal death occurs when an infant is born alive but dies within 28 complete days [3]. Perinatal mortality combines stillbirths with early neonatal deaths (within a week of birth), and late neonatal mortality occurs after seven days but within 28 days. As under-five and infant mortality rates fall, early deaths become proportionately greater contributors to global child mortality. India’s most recent annual estimate of under-five deaths is 1.8 million [1], a drop from about 2.4 million in 2000 [4], of which about 1.0 million are newborn deaths. Early neonatal deaths now make up four-fifths of neonatal deaths and infant mortality three-quarters of under-five mortality [4].

The three major causes of newborn deaths are infection (often classified as sepsis or pneumonia), complications of preterm birth, and birth asphyxia (the aetiology of failure to breathe at birth is often unclear, and the currently recommended term is ‘intrapartum-related death’) [2]. Neonatal mortality rates (NMRs) are decreasing, albeit at a rate that might not reach the targeted two-thirds reduction set by UN Millennium Development Goal 4 (http://www.un.org/millenniumgoals/childhealth.shtml). India’s NMR fell from 49 to 39 per thousand live births between the early 1990s and the early 2000s [5]. Decreasing mortality rates imply changing contributions of individual causes. Recent global estimates put prematurity first (28-30%), followed by infection (25-28%) and asphyxia (23-24%) [1,6,7]. In India, sepsis and pneumonia accounted for 52% of neonatal deaths in rural Maharashtra in the early 2000s [8]. Recent estimates for India suggest that sepsis or pneumonia underlie 30% of newborn deaths, birth asphyxia 20% and preterm 17% [4].

Such figures are generally based on verbal autopsy, in which interviews with the deceased’s caregivers are examined to ascribe cause of death [9-11]. Infant and child verbal autopsy tools have been developed over the last twenty years [12-18], and used in a range of settings [19-22]. There are several possible approaches to data collection. The interviewer (often with a non-medical background) may discuss the events that preceded death with a child’s mother or family and record the narrative in open-ended form. Alternatively, a series of closed questions may be asked, designed to establish the presence or absence of specific signs and symptoms, or particular health care actions [23,24]. A clinician then classifies the cause of death, with or without the assistance of algorithms [25-28]. India’s Million Death Study employs an enhanced verbal autopsy embedded in its national sample registration system (SRS) [29]. Delays in treating perinatal complications are important contributors to morbidity and mortality. Even minor delays in providing emergency obstetric care around the time of birth can be significant [2]. Broadly, delays occur at three stages: (1) the decision to seek care, (2) reaching a health facility, and (3) the provision of adequate care [30]. Although the ‘three delays’ model was developed to understand maternal mortality, it has recently been applied to neonatal and perinatal deaths [31,32].

India is urbanising and the growth rate in slum areas is almost twice that of urban areas as a whole. Slums are typically compact areas with poorly-built, congested housing, unhygienic environmental conditions, and inadequate infrastructure, sanitation and drinking water facilities. In most Indian cities – including Mumbai – poverty is more prevalent in slums and most poor people live in slum areas [33]. Healthcare is provided by a diversity of public and private providers. The Municipal Corporation of Greater Mumbai (MCGM) provides more than one-quarter of the city’s approximately 40,000 available beds and is a major healthcare provider for the poor [34]. The extensive private sector includes a large number of individual practitioners who offer limited services.

There are some data on urban mortality rates in low- and middle-income countries [35-37], but there is little information on patterns of cause-specific mortality for stillbirths and neonatal deaths in urban settings [35,38,39]. Uptake of antenatal care and institutional delivery is generally higher than in rural areas, and mortality rates lower. In Mumbai, for example, attendance for three or more antenatal consultations and institutional delivery run at about 90% [40], although socioeconomic inequalities mean that poorer families have higher stillbirth and neonatal mortality rates than wealthier families [41].

Mumbai’s City Initiative for Newborn Health aimed to improve the experience and outcomes of maternity for poorer women living in slum settlements. In a randomized controlled trial of community women’s groups, local facilitators helped women make appropriate choices for their maternity and childcare. The model was based on similar programmes which have suggested that improvements in newborn survival are possible through community mobilisation [42,43].

Our objectives in this paper were to classify stillbirths and neonatal deaths according to international cause-specific criteria and to identify major causes of delay in seeking and receiving health care for maternal and newborn health problems. We examined the causes of death as a test of three hypotheses: that early neonatal deaths would make up a greater proportion of neonatal mortality than in rural settings; that there would be fewer deaths from infection, which dominate late neonatal mortality; and that the proportions of intrapartum-related and preterm deaths would be relatively higher.

## Methods

The study used data collected by a maternal and child vital registration system in a population of 283 000 covering 48 slum localities in six municipal wards [44]. There were eight randomly selected localities per ward, each with about 1200 households and a population of around 6000. Localities acted as clusters within a cluster randomized controlled trial of community mobilization as a means to improve public health outcomes [44]. Data collection involved prospective identification of births and birth outcomes by 99 locally resident women (generally two per cluster), covering about 600 households each, who reported weekly to one of 12 interviewers. The interviewers confirmed births by visiting families at home and arranging a repeat visit for interview at about six weeks after delivery. These interviews included demographic information, obstetric history and details of care in the index pregnancy. In the event of a maternal, infant or child death, one of six supervisors accompanied the interviewer on a condolence visit and completed a verbal autopsy with close family members.

We used a verbal autopsy tool with both open and closed questions based on versions from partner projects in Nepal, Bangladesh, Malawi and India, and on serial drafts that developed into the current WHO standard (http://www.who.int/whosis/mort/verbalautopsystandards/en/index.html).[45,46], The tool included a structured narrative section in which mothers and families were encouraged to describe their experiences of pregnancy and delivery, illnesses and care-seeking, and the circumstances surrounding the death. Completed verbal autopsies were checked at weekly meetings with project officers. Quantitative data were entered into electronic relational database management systems in Microsoft Access (Microsoft Corporation), with validation constraints and enforced referential integrity. The interviewers manually documented narratives, which were subsequently transcribed and translated into English. Participants gave verbal consent to interview, data were anonymised, and no identifiable information appeared in output.

Table 1 summarizes the cause of death classification. Because of a need to spread workload, verbal autopsy questionnaires were assessed by five practising clinicians. Most were assessed by two (144, 65%), 28 by three, and five by four. A single cause of death was assigned for each infant. Agreement at first pass was achieved in 109/221 cases (49%). When clinicians did not agree, the classification was adjudicated by a paediatrician experienced in verbal autopsy classification (DO). The overall *kappa* statistic for agreement between physicians was 0.65 (95% CI 0.615 - 0.732).

**Table 1:** Classification system for causes of stillbirth and neonatal death

**Neonatal death**	**Antepartum/macerated stillbirth**
Early or late	Intrapartum/fresh stillbirth
Preterm or term	
	1. Congenital anomalies
1. Congenital anomalies	2. Accident or external condition
2. Accident or external condition	3. Associated with obstetric complications
3. Asphyxia	4. Prematurity
3a. Asphyxia associated with obstetric complications	5. Multiple pregnancy
4. Prematurity	6. Other
5. Severe infection	7. Unclassifiable
5a. Neonatal tetanus	
6. Other	
7. Unclassifiable	

Quantitative analyses were done in Stata 11 (Stata Corporation, USA). We compared women who had delivered in Mumbai and suffered bereavement (either stillbirth or neonatal death: technically, extended perinatal mortality) with women whose babies had survived. We used multivariable logistic regression, including a random effect for cluster to account for the collection of data from discrete slum localities. Quadrature checks supported the applicability of this approach. Covariates forced into the model included a continuous variable describing maternal age, and indicator variables describing each category of education, religion, duration of residence, socioeconomic status, parity, previous stillbirth, number of antenatal consultations, and site of delivery. Socioeconomic status was based on quintiles of an asset index derived from the first component of a principal components analysis [47]. We repeated the analysis twice using the same covariates, but with either stillbirth or neonatal death as the outcome. Women whose babies were stillborn were compared with women whose babies were live-born (although these could still have suffered a neonatal death). Women whose babies died in the neonatal period were compared with women whose babies survived.

We did a content analysis of qualitative narratives based on the three delays model to classify and quantify delays, and to document their underlying causes. Since more than one delay per death was possible, we considered each instance of delay separately. Two non-clinical researchers (UB and GA) and an assistant researcher reviewed transcripts independently for common themes and evidence of delay. Delays were classified as ‘delay 1’, ‘delay 2’ or ‘delay 3’, subcategorised according to the main causes and recorded in Microsoft Excel (Microsoft Corporation). Independent classifications and subcategories were then compared and each narrative discussed to consensus. Validation was carried out by crosschecking selected cases against the original transcripts.

Data for the study originated from the trial approved by the Municipal Corporation of Greater Mumbai and the Independent Ethics Committee for Research on Human Subjects (Mumbai committee, reference IEC/06/31) [44].

## Results

**Figure 1:**
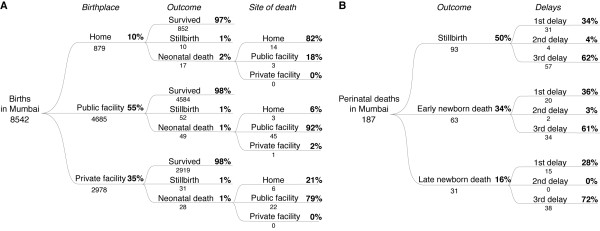
Study profile.

Figure 1 summarises births, stillbirths and neonatal deaths. In two years, 1^st^ October 2005 – 30^th^ September 2007, we identified and confirmed 13 467 births, representing an annual crude birth rate of 23.8 per 1000. We are certain of the survival status of 11 464 infants. There were 159 stillbirths, 11 305 live births, and 210 newborn deaths. The stillbirth rate was 13.9 (95% CI 11.8-16.2) per 1000 births and the NMR 18.6 (16.2-21.2) per 1000 live births. Verbal autopsies were completed for 105/159 stillbirths (66%) and 116/210 neonatal deaths (55%). The main reasons for loss to follow-up were relocation or the fact that women who lived elsewhere had only come to the area for delivery. Of the neonatal deaths, 87 were early (75%); 40 (34%) occurred within one day of birth, 12 (10%) within two days, and 11 (9%) within three. Male infants made up 54% of stillbirths (57/105) and 59% of neonatal deaths (67/114).

Table 2 summarises the agreed causes of death. Of 105 stillbirths, 65 were fresh (62%). Obstetric complications dominated the cause classification. Combining the categories for asphyxia and ‘asphyxia with obstetric complication’ as an indicator of intrapartum-related neonatal death, the main causes of neonatal death were intrapartum-related (28%), prematurity (23%), and severe infection (22%). The pattern of causes was internally plausible in that intrapartum-related and prematurity were dominant causes of early neonatal mortality, while severe infection dominated late neonatal mortality. Congenital malformations explained 6% of deaths. Combining fresh stillbirths with intrapartum-related neonatal deaths, the estimated contribution of intrapartum-related deaths to perinatal mortality was 50%.

**Table 2:** Causes of stillbirth and newborn death, based on clinician review of verbal autopsy

**Stillbirth**	**Fresh**		**All**			
	Count	(%)	Count	(%)		
Associated with obstetric complication	41	(63)	50	(48)		
Multiple pregnancy	3	(5)	9	(8)		
Prematurity	4	(6)	4	(4)		
Accident or external condition	2	(3)	3	(3)		
Congenital anomalies	1	(2)	3	(3)		
Other	2	(3)	4	(4)		
Unclassifiable	12	(18)	32	(30)		
Total	65	(100)	105	(100)		
Neonatal death					All	
	Count	(%)	Count	(%)	Count	(%)
Asphyxia	21	(24)	0	(0)	21	(18)
Asphyxia associated with obstetric complication	11	(13)	1	(3)	12	(10)
Prematurity	27	(31)	0	(0)	27	(23)
Severe infection	5	(6)	20	(69)	25	(22)
Congenital anomalies	5	(6)	2	(7)	7	(6)
Other	10	(11)	0	(0)	10	(9)
Unclassifiable	8	(9)	6	(21)	14	(12)
Total	87	(100)	29	(100)	116	(100)

To examine characteristics of bereaved mothers and sites of delivery and death, we limited the dataset to 8542 births that had taken place within Mumbai. These included 93 stillbirths, 63 early neonatal deaths and 31 late neonatal deaths. Table 3 compares the characteristics of bereaved and non-bereaved mothers and provides adjusted odds ratios from a multivariable model (aORs). Bereavement was not associated with maternal age, education, religion, duration of residence, parity, or site of delivery. It was strongly associated with the occurrence of a previous stillbirth, and also with socio-economic status and number of antenatal care visits when these factors were included in the model as ordered categorical variables. We repeated the analysis using stillbirth and neonatal death as outcomes instead of bereavement. The effect of the independent variables was relatively unchanged, except previous stillbirth, which was associated with greater odds of stillbirth (aOR 7.69, 95% CI 4.47-13.24) than of neonatal death (aOR 3.60, 95% CI 1.83-7.07) (data not shown).

**Table 3:** Characteristics of non-bereaved and bereaved mothers who delivered in Mumbai, 2005-2007

	**Bereaved mothers**		**Non-bereaved mothers**			
	n	(%)	n	(%)	aOR	95% CI
Mean age (SD)	25.2	(4.4)	24.6	(4.4)	1.00	0.96-1.04
Education*						
None or informal	61	(33)	2337	(28)	1.00	
Primary	16	(9)	541	(6)	1.39	0.78-2.46
Secondary	98	(52)	4822	(58)	1.16	0.80-1.68
Higher	12	(6)	648	(8)	1.23	0.62-2.45
Religion						
Hindu	84	(45)	3727	(44)	1.00	
Muslim	91	(49)	4063	(49)	0.87	0.62-1.22
Other	12	(6)	565	(7)	1.00	0.53-1.90
Time living in community						
Less than 1 year	24	(13)	1243	(15)	1.00	
1-4 years	71	(38)	3581	(43)	1.07	0.66-1.72
5-10 years	50	(27)	1899	(23)	1.32	0.78-2.23
More than 10 yrs	42	(22)	1632	(19)	1.38	0.81-2.34
Socioeconomic quintile						
Lowest	44	(23)	1566	(18)	1.00	
Next to lowest	48	(26)	1667	(20)	1.00	0.65-1.53
Middle	46	(25)	1651	(20)	1.01	0.65-1.58
Next to highest	18	(10)	1735	(21)	0.39	0.22-0.70
Highest	31	(16)	1736	(21)	0.69	0.41-1.16
Parity						
1	43	(23)	2556	(30)	1.00	
2	47	(25)	2331	(28)	0.94	0.61-1.47
3	38	(20)	1575	(19)	0.98	0.59-1.63
4	28	(15)	922	(11)	1.15	0.64-2.06
5 or more	31	(17)	971	(12)	1.00	0.51-1.94
Previous stillbirth						
No	155	(83)	8073	(97)	1.00	
Yes	32	(17)	282	(3)	5.49	3.58-8.42
Antenatal consultations						
0	27	(14)	320	(4)	1.00	
1 or 2	14	(7)	351	(4)	0.44	0.22-0.88
3 or more	146	(78)	7684	(92)	0.23	0.14-0.37
Site of delivery						
Home	27	(14)	852	(10)	1.00	
Public hospital	101	(54)	4584	(55)	1.35	0.81-2.27
Private hospital	59	(32)	2919	(35)	1.29	0.75-2.22
Total number of women	187	(100)	8355	(100)		

SD: standard deviation. aOR: Adjusted odds ratio for bereavement from random effects logistic regression model, including maternal age as a continuous variable, and maternal education, religion, duration of residence, socio-economic quintile, parity, previous stillbirth, antenatal care visits, and site of delivery as categorical variables.

CI: confidence interval

* 7 cases missing.

**Figure 2:**
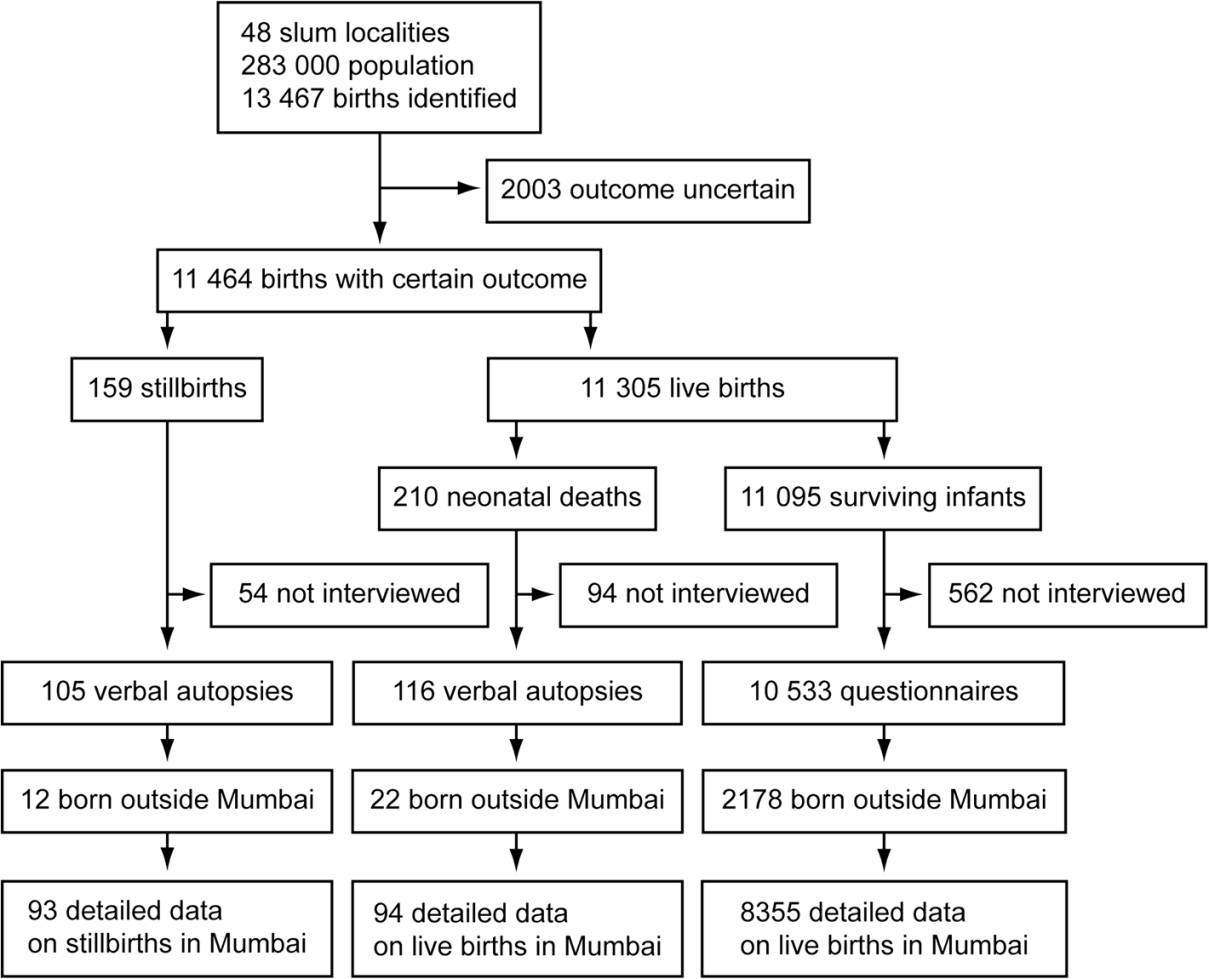
**Pathways to mortality.** (**A**) Birthplace, outcome and site of death. (**B**) Delays associated with stillbirth, early and late neonatal death. For 8542 births in Mumbai, 2005–2007.

We examined sites of delivery and death, again limited to births in Mumbai, 90% of which were institutional (Figure 2A). The commonest delivery site was the public sector (55%), followed by the private sector (35%) and home birth (10%). Outcomes were similar across delivery sites. The commonest site of neonatal death after a home delivery was the home (82%). Of neonatal deaths of infants born in the public sector, 92% occurred in public sector facilities; 79% of neonatal deaths of infants born in the private sector were also at public sector facilities. Only one neonate died in the private sector.

We identified delays in 121/187 (65%) narratives. In 70 (38%) only one delay was recorded, in 28 (15%) two delays, and in 23 (12%) three or more. In total, we documented 201 individual delays: 66 delay 1; 6 delay 2; and 129 delay 3 (Figure 2B). There were more delays than deaths because multiple delays were possible.

The narratives revealed a variety of interrelated factors that contributed to delays in deciding to seek care and receiving treatment. The main reasons for not seeking prompt care for both maternal and newborn complications were a failure to recognise symptoms or their potential severity. In 11 narratives, families had attempted to treat the condition at home. This was more common for newborns than for mothers, and we classified them as a delay because we felt that the death might have been avoided if institutional care had been sought. Less common reasons included a lack of money, having nobody to take care of the children, and being unable to decide in the absence of the husband or a family member. We examined families’ choice of health care provider for degrees of illness by comparing initial care-seeking sites for sick newborns who survived with those who did not. The commonest sites for newborns who survived were individual private practitioners, followed by private hospitals; newborns who later died were first taken to municipal hospitals, followed by private hospitals and tertiary facilities.

A major cause of delay in receiving medical care was referral between health facilities. Of 68 documented referrals, 47 were of neonates and 21 of mothers. Most referrals for maternal complications were from private hospitals and municipal general hospitals to a tertiary hospital. Most newborn referrals occurred between private hospitals, although municipal general hospitals and private providers also referred upwards to tertiary facilities. Five referrals were between tertiary hospitals. 12 cases involved multiple referrals, most of which were of newborns. In two-thirds of all referrals, we felt that the delays directly contributed to the stillbirth or neonatal death.

In 41 narratives, where information was available, the main reported reasons for referral were a lack of equipment or services at the facility, clinical indication (i.e. conditions requiring specialised care) and, less commonly, the absence or unavailability of health personnel. In 15 narratives, respondents reported being refused admission to a health facility. These were mostly women who had presented at municipal hospitals in early stages of labour and were told to return closer to their delivery time. In six narratives, families abandoned one health facility for another, usually because they were dissatisfied with the level of care or could not afford treatment.

Criticisms of the healthcare system included excessive waiting times, provider apathy or negligence, inappropriate treatment, and poor communication. None of the bereaved families had reported their complaints, mainly because they lacked faith in the redressal system. Some bereaved mothers felt responsible for the death, because they had been careless with their own health, had not attended regular antenatal consultations or sought care for ill health, had not complied with health advice, or had not recognised and responded to their child’s illness promptly enough. An example narrative follows.

"Ashifa (name changed) started antenatal care in her second month of pregnancy with a local private provider and, from the seventh month, at a municipal maternity home. One evening, she started feeling light abdominal pain. A friend advised her to go to the maternity home, but she felt that her delivery was not yet due and needed to wait for her husband to arrive home. Later, the pain became severe and her waters broke. At about 4 a.m. her husband and her friend took her to the maternity home. The examining doctor said that both she and the baby were ‘at risk’ and referred them to a municipal hospital."

"Ashifa and her husband felt that the service in the hospital was inefficient and decided to go to a private nursing home instead, where she gave birth there to a baby girl at about 8 a.m. Because her delivery was normal, they felt that the doctor at the municipal maternity home had unnecessarily wanted to refer her. Ashifa was jaundiced and weak. She was not allowed to feed her baby and was told to go to another hospital because the private provider did not have the necessary facilities for treatment."

"They went to a private hospital and Ashifa was admitted. Mother and baby were kept apart during her hospitalisation; the nurse showed Ashifa the baby every day but did not tell her what they were feeding her. Ashifa was discharged after 12 days with one-month’s supply of medicines and was told to feed her baby infant formula. After a further 15 days she started breastfeeding in addition to the formula. About four days later her baby developed a fever, breathing problems and a nosebleed. They immediately took her back to the private nursing home where she delivered but were referred to the municipal hospital. They did not go, but returned home where the baby’s condition deteriorated. Their neighbours advised them not to go to the hospital, saying the baby was already dead and that they would have problems claiming the body. Ashifa and her husband decided not to consult another doctor, and, later, buried the baby themselves."

## Discussion

The clinical findings were consistent with our hypotheses. In Mumbai’s slum settlements, home to more than half of the city, early neonatal deaths made up 75% of neonatal deaths, intrapartum-related deaths were the greatest cause of neonatal mortality, and severe infection was the third commonest cause. Intrapartum-related deaths accounted for half of perinatal mortality. Bereaved women tended to be poorer, had fewer antenatal consultations and were more likely to have suffered previous stillbirth than non-bereaved mothers. We have previously shown associations between socioeconomic status and health outcomes in the same population [48]. Although uptake of antenatal care can be an indicator of quality in health facilities, it is difficult to know whether the association of infant survival with the number of antenatal care visits was the result of the care itself or the tendency for women at lower risk to attend check-ups more assiduously. Delays were identified in two-thirds of narratives, were predominantly related to the provision of care, and were mostly attributable to referrals between health facilities. These were substantially higher than in our previous research on care-seeking for a range of maternal morbidities [49].

The main limitations of the study were, predictably, recruitment and the shortcomings of the verbal autopsy method. Follow-up was difficult. We have documented a 25% turnover in households in the slum settlements covered by the study. Combined with the trauma of bereavement, which led some families to relocate, we achieved an interview proportion of between a half and two-thirds of stillbirths and neonatal deaths. We do not know if the causal structure of mortality was different in families who were not interviewed. Verbal autopsy is based on lay recall of events. Because there is no gold standard with which to compare the findings, misclassification of causes of death is possible [50]. We were unable to triangulate our findings with hospital records and municipal data because of the complexity of matching across multiple private and public sector hospitals and the vagaries of certification. It is possible that parents’ recall led to over-ascription of obstetric complications and perhaps overestimation of the culpability of healthcare workers. The major limitations of the classification process were our use of a single cause of death, which could not account for co-morbidity, and the acceptable but limited agreement between physicians. The largest number of mismatches (21) involved differentiation between asphyxia and asphyxia combined with obstetric complications. The narrative data were insufficient to fully explore the complex, interrelated factors that influenced care-seeking decisions and the provision of healthcare

Incomplete accounts of events made it difficult to classify delays (for example, not admitting a woman in early stages of labour). Since we limited our analysis to bereaved families, the findings cannot be generalised to those who did not experience delays or to non-bereaved families. However, we believe that this is the first study to apply the three delays framework to perinatal deaths in urban India.

The ability of families to recognise the symptoms and severity of complications was an important precursor to healthcare decisions. Although similar research has suggested that poor recognition of some symptoms of maternal and infant illness does not necessarily limit the use of health services [51-53], it has been considered an important preventable factor in maternal deaths in Mumbai [54]. These, however, form only part of a broad, complex range of determinants and interactions that influence care-seeking behaviour. Most families sought care for maternal and newborn complications, indicating a perceived need and preference for institutional health care. In previous research we have shown high levels of care-seeking within 48 hours for all types of maternal morbidity [49]. However, some families, at least, did not identify serious symptoms and the need for urgent care. Community-based health education and mobilisation strategies need to work with the most susceptible families, especially the poor, low users of health services, and those with a history of maternal complications.

Of concern is the capacity of the health system to respond efficiently and appropriately to life-threatening health conditions. In the public sector, users complain of shortages of staff and equipment and impolite treatment [55,56]. The private sector in India is virtually unregulated and many private practitioners lack formal training [53,57,58]. We were struck by the number of referrals between health providers and their contribution to deaths. While inappropriate care-seeking is a possibility, our finding that families tended to seek treatment for serious newborn illness at higher-level facilities implies that they are able to make rational assessments about the level of care required. That many referrals were from private hospitals corroborates claims that private providers lack the capacity to manage serious illness [53,57,58]. At the same time, peripheral public hospitals lack emergency care facilities and are also major referrers [59].

Our finding that most referrals were not clinical, that some were between tertiary facilities, and that most deaths occurred in public hospitals, are at least suggestive of deficiencies in quality and infrastructure and a system poorly organized to prioritize and manage emergencies [60,61]. Tertiary care providers are often at the receiving end of inappropriate or delayed treatment and referrals: higher maternal mortality rates in major referral hospitals in Mumbai have been attributed to the large number of complicated referrals they receive [54,59,62]. At best, the absence of a well-functioning referral system adds to overcrowding in these hospitals [63]; at worst, it leads some to abandon the healthcare system altogether.

In the metropolitan setting with high uptake of healthcare and wide availability of medication, the health of mothers and infants during and shortly after delivery is a priority. Quicker identification of problems, protocolized management, and rapid – systematic – transfer to an appropriate facility are key aims of the work that we are doing with the Municipal Corporation of Greater Mumbai.

## Conclusions

Although neonatal mortality rates in India are in decline, more can be done to achieve the target set by MDG 4. In urban Mumbai, the problem is not finding a health provider, but understanding the factors that influence care-seeking decisions for serious maternal and newborn illness and, once at a health facility, ensuring that rapid and appropriate care is provided. Further research is needed to explore how poor urban families assess symptoms and their severity, how this relates to other factors, such as opinions about providers and family support, and how they decide at which point health care is necessary. Challenges to the health system include better regulation of private providers, improving the capacity of peripheral facilities to manage serious complications, and ensuring a well-functioning referral system between and within sectors, including clear protocols for care and transfer at each level of the health system and communication between facilities. We also support the introduction of mechanisms to improve provider accountability and regular institutional perinatal audit.

## Competing interests

The authors declare that they have no competing interests. UB, GA and DO had full access to all the data in the study and had final responsibility for the decision to submit for publication.

## Authors’ contributions

UB, GA and DO conceived the study. UB and SD supervised data collection, entry, and cleaning. WJ and DO classified verbal autopsies. UB and GA analysed qualitative data and GA and DO analysed quantitative data. UB, GA and DO co-wrote the manuscript. NSM coordinated the project and WJ had overall responsibility for SNEHA research activities. All authors contributed to critique and modification of the manuscript and read and approved the final version.

## Role of the funding source

The City Initiative for Newborn Health was supported by the ICICI Centre for Child Health and Nutrition and by The Wellcome Trust (Grant number 081052). The sponsors had no role in the study design, data collection, analysis, interpretation or writing of the article.

## Pre-publication history

The pre-publication history for this paper can be accessed here:

http://www.biomedcentral.com/1471-2393/12/39/prepub
